# The effect of two pre-cryopreservation single layer colloidal centrifugation protocols in combination with different freezing extenders on the fragmentation dynamics of thawed equine sperm DNA

**DOI:** 10.1186/1751-0147-54-72

**Published:** 2012-12-05

**Authors:** Luna Gutiérrez-Cepeda, Álvaro Fernández, Francisco Crespo, Miguel Ángel Ramírez, Jaime Gosálvez, Consuelo Serres

**Affiliations:** 1 Animal Medicine and Surgery Deparment, Veterinary Faculty, UCM, Avda. Puerta de Hierro s/n, Ciudad Universitaria, 28040, Madrid, Spain; 2 Centro Militar de Cría Caballar, (FESCCR-Ministerio de Defensa), Ávila, Spain; 3 Halotech DNA, S.L. UAM, Madrid, Spain; 4 Biology Department, Genetic Unity, UAM, Madrid, Spain

**Keywords:** Colloidal centrifugation, Equine sperm, DNA fragmentation, Cryopreservation, Equipure®

## Abstract

**Background:**

Variability among stallions in terms of semen cryopreservation quality renders it difficult to arrive at a standardized cryopreservation method. Different extenders and processing techniques (such us colloidal centrifugation) are used in order to optimize post-thaw sperm quality. Sperm chromatin integrity analysis is an effective tool for assessing such quality. The aim of the present study was to compare the effect of two single layer colloidal centrifugation protocols (prior to cryopreservation) in combination with three commercial freezing extenders on the post-thaw chromatin integrity of equine sperm samples at different post-thaw incubation (37°C) times (i.e., their DNA fragmentation dynamics).

**Results:**

Post-thaw DNA fragmentation levels in semen samples subjected to either of the colloidal centrifugation protocols were significantly lower (p<0.05) immediately after thawing and after 4 h of incubation at 37°C compared to samples that underwent standard (control) centrifugation. The use of InraFreeze® extender was associated with significantly less DNA fragmentation than the use of Botu-Crio® extender at 6 h of incubation, and than the use of either Botu-Crio® or Gent® extender at 24 h of incubation (p<0.05).

**Conclusions:**

These results suggest that single layer colloidal centrifugation performed with extended or raw semen prior to cryopreservation reduces DNA fragmentation during the first four hours after thawing. Further studies are needed to determine the influence of freezing extenders on equine sperm DNA fragmentation dynamics.

## Background

The use of cryopreserved equine sperm is becoming increasingly important in breeding strategies. However, variability among stallions in terms of semen cryopreservation quality renders it difficult to arrive at a standardized cryopreservation method [1,2]. Neither are there standard methods for determining pre-cryopreservation nor post-thaw sperm quality, nor indeed are there any standard artificial insemination protocols [3].

Traditional laboratory methods are unable to accurately determine the fertility of cryopreserved semen [4]. Spermatozoa with damaged chromatin appear normal in terms of their membrane integrity, morphology and motility, but their use would lead to post-fertilization embryo failure [5]. The need to include sperm chromatin integrity analysis in the assessment of semen has been indicated by several authors who report a direct correlation between fertility and sperm chromatin integrity [6] but a poor correlation between fertility and the classically measured sperm quality variables [7-10]. The analysis of DNA fragmentation dynamics is important since DNA can become fragmented during this time, even within the female reproductive tract.

Certainly, López-Fernández et al. [9] and Cortés-Gutiérrez et al. [11] have shown, at least *in vitro*, that the DNA of thawed semen incubated at 37°C becomes less stable over time. Moreover, it is important the use of the dynamic form of the Sperm DNA Fragmentation (SDF) assay for evaluating centrifugation and / or other ex vivo procedures, as a single basal assessment of SDF may inadvertently result in a false-positive evaluation of DNA quality [6].

The wide variability among stallions in terms of how well their semen can be cryopreserved demands that test freezing be performed to determine which technique is most suitable in each case [12]. This is particularly important when dealing with “bad freezer stallions”, a significant problem among Purebred Spanish horses.

The pre-cryopreservation colloidal centrifugation of equine semen can increase the post-thaw number of sperm cells with normal morphology, as well as improve sperm motility (and sperm motility characteristics), membrane integrity and viability [6,13-18]. Certainly, colloidal centrifugation selects spermatozoa with intact and mature chromatin, which have a higher density than immature or damaged sperm [19-21]; further, the removal of damaged or dead spermatozoa and leukocytes may help maintain sperm chromatin integrity by preventing future damage caused by reactive oxygen species (ROS) [10,19,20]. The advantages of such centrifugation become more evident when semen is preserved cooled or in a state of cryopreservation [20]. Colloidal centrifugation can also eliminate pathogens from semen [22-26].

Although the use of colloidal centrifugation has been correlated with lower sperm DNA fragmentation levels in fresh, cooled [10,19,20,25,27] and cryopreserved semen (with centrifugation performed before or after the cryopreservation step) [17,28,29], few studies have examined the dynamics of DNA fragmentation. To our knowledge this is the first study that combines the effect of colloidal centrifugation prior to cryopreservation and equine freezing extenders on the dynamics of DNA fragmentation over the post-thaw time.

The aim of the present study was to compare the effect of two single layer colloidal centrifugation protocols (prior to cryopreservation), developed at our laboratory [21] in combination with three commercial freezing extenders on the post-thaw chromatin integrity of equine sperm samples at different post-thaw incubation (37°C) times (i.e., the fragmentation dynamics of the sperm DNA).

## Methods

### Animals

Seventeen ejaculates (2–3 per stallion) were obtained between January and March 2010 from six Purebred Spanish horses at the *Depósito de Sementales de Ávila* (FESCCR) (40.66ºN 4.70ºW), Spain. The donor stallions, all between 7 and 15 years of age, were clinically healthy and of documented fertility. The diets and housing of these horses were those deemed to keep them in optimum condition.

### Semen collection and pre-cryopreservation analysis

Semen was collected by allowing the stallions to mount a phantom, using a Missouri-model artificial vagina (Nasco, Fort Atkinson, WI, USA) warmed to 45-50°C and lubricated with a sterile non-spermicidal gel (IMV Technologies, L’Aigle, France). A mare in oestrous was used to induce sexual activity. After stabilization of the extragonadal sperm reserves (daily collection) semen was collected on a regular basis (two collections/week).

The gel-free ejaculates (maintained at 37°C) were evaluated for volume using a graduated test tube, sperm motility and concentration using a computerized analysis system running ISAS® software [PROYSER, Madrid, Spain].

### Semen centrifugation

**Figure 1:**
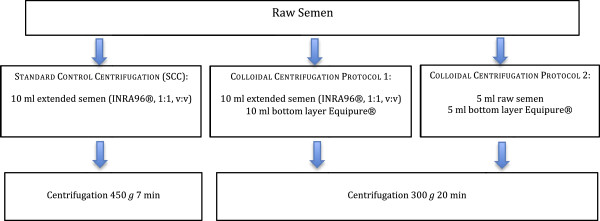
Semen centrifugation protocols.

Samples of all ejaculates were subjected to each of the following treatments (Figure 1).

● *Standard control centrifugation (SCC):* 10 ml of semen extended 1:1 v:v with Inra96® (IMV technologies, L’Aigle, France) were placed in a 50 ml Falcon tube and centrifuged at 450 *g* for 7 min.

● *Colloidal centrifugation Protocol 1:* 10 ml of semen extended 1:1 v:v with Inra96® were placed on top of a 10 ml layer of Bottom Layer® EquiPure® [Nidacon, International AB, Mölndal, Sweden] equilibrated at 22°C in a 50 ml Falcon tube. Care was taken to avoid mixing the sperm and colloid phases. The loaded tubes were centrifuged at 300 *g* for 20 min.

● *Colloidal centrifugation Protocol 2:* 5 ml of raw semen (i.e., without extender) were placed over 5 ml of Bottom Layer® EquiPure® following the same instructions as in Protocol 1.

● After centrifugation, the supernatant (semen extender, seminal plasma and colloid) was removed by aspiration. The resulting sperm pellets were resuspended for cryopreservation in the presence of different extenders.

### Cryopreservation with different extenders

**Figure 2:**
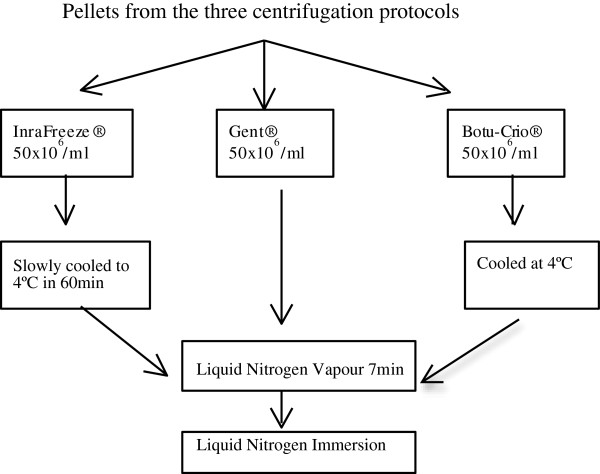
Addition of post-centrifugation pellets to extenders, and cryopreservation.

The required volume of each pellet was taken to obtain a final sperm concentration of 50 × 10^6^ ml^-1^ in 5 ml of each of three commercial freezing extenders: Gent® (Minitüb®, Abfullund Labortechnik GmbH & Co., Tiefenbach, Germany), InraFreeze® (IMV technologies, L’Aigle, France) and Botu-Crio® (BioTech, Botucatu, Sao Paulo, Brazil) (total number of treatments therefore = 9; see Figure 2). After resuspension, the semen was packed into 0.5 ml polyvinyl chloride straws (IMV International, St Paul, MN, USA). The straws containing sperm extended with Gent® (G) extender were frozen without prior cooling [30,31]. Those containing sperm extended with InraFreeze® (I) were frozen after being slowly cooled to 4°C at a rate of 0.3°C/min, as recommended by the manufacturer. Finally, those containing sperm extended with Botu-Crio® (B) were frozen after being cooled at 4-6°C for 20 min, as recommended by the manufacturer. In all cases the straws were frozen horizontally in racks placed 4 cm above the surface of liquid nitrogen for 7 min, after which they were plunged into this liquid nitrogen as described by Cochran et al. [30].

### Assessment of post-thaw sperm chromatin integrity

After 4 weeks of storage the straws were thawed by immersion in a water bath at 37°C for 1 min. Sperm chromatin integrity was assessed via the sperm chromatin dispersion test [9] making use of the Equus-Halomax® Kit (Halotech DNA, SL., Madrid, Spain). The degree of DNA damage in each sample was quantified using the sperm DNA fragmentation index (sDFI), which reflects the percentage of cells with fragmented DNA.

An aliquot of sperm from each straw was diluted to 10 × 10^6^ ml^-1^ and processed following the kit instructions. DNA damage was visualized using a Proyser fluorescent microscope (Proyser, Madrid, Spain) employing SyberGreen II fluorochrome at 40× concentration. Sperm heads showing a large halo of chromatin dispersion were understood to contain highly fragmented DNA. The percentage of spermatozoa with such fragmented DNA was calculated and expressed as a percentage of the total sperm count. Observations were made at 0, 4, 6 and 24 h of incubation in a 37°C water bath.

### Statistical analysis

ANOVA and DUNCAN test were used to compare sDFI values. Repeat measures analysis (with Greenhouse correction) was performed to follow the change in sDFI over incubation time (within-subjects factor), considering the variable ‘donor’ as a source of variation (between-subjects factor). The influence of the interaction *incubation time × donor* on sDFI was also examined. Significance was set at p<0.05. Data were processed using the IBM® SPSS-19® statistical package.

## Results

Media sperm concentration value in fresh semen was 273,35 × 10^6^ spermatozoa/ml (range between 189,9 and 361 × 10^6^ spermazoa/ml). After centrifugation, the average sperm concentration of the colloidal-centrifuged pellets (Protocols 1 and 2) was approximately half that of the SCC pellets (544.5 million sperm/ml and a 57.79% sperm yield vs. 928 million sperm/ml and a 98.5% sperm yield).

Significant differences were seen between stallion donors in terms of mean sDFI (p<0.05). Two groups with significantly different mean sDFI values were established: low (<20.23%) and high (>27.13%) (Table 1).

**Table 1:** sDFI value groups (means±SD)

**STALLION**	**Sperm DNA fragmentation index (sDFI)**	
	**Low average levels**	**High average levels**
B	15.8135^b^±8,88	
C	18.3176^b^±9,34	
F	19.4167^b^±13,95	
A	20.2345^b^±12,73	
D		27.1280^a^±13,20
E		30.9288^a^±14,97

Letters A-F represent the different stallions. Letters in superscript represent significant differences in average sDFI values (p<0.05), revealing two groups of stallions.

**Figure 3:**
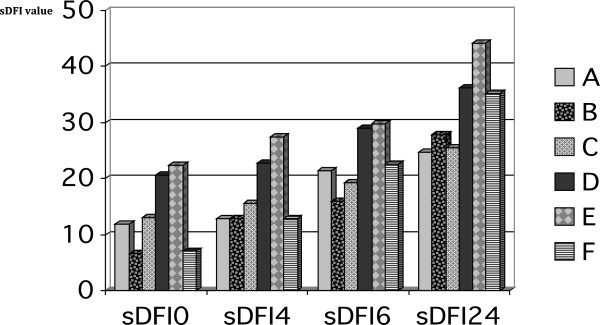
**DNA fragmentation values (sDFI) over incubation time for the different stallions.** Legend: Average DNA fragmentation index values immediately post-thawing (sDFI 0) and after 4 (sDFI 4), 6 (sDFI 6) and 24 (sDFI 24) hours of incubation. Letters (A-F) represent the different stallions. sDFI values increased significantly with time (p<0.05), but not equally so across all the stallions (p<0.05).

The sDFI values increased over incubation time for each ejaculate in all nine treatments (Table 2). However, this increase was different in different donors, with the interaction *incubation time x donor* having a significant effect on the sDFI value (p<0.05) (Figure 3).

**Table 2:** Average DNA fragmentation values associated with the centrifugation+extender treatments (means±SD)

**Treatment**	**sDFI 0**	**sDFI 4**	**sDFI 6**	**sDFI 24**
B-C	16.36^a^±11,56	22.15^b^±11,58	27.01^a^±15,66	38.40^b. c^±13,23
B-1	13.23^a^±7,08	16.06^a. b^±5,64	27.07^a^±7,37	35.95^a. b. c^±10,38
B-2	11.86^a^±5,87	18.11^a. b^±7,32	23.13^a^±8,62	33.91^a. b. c^±14,50
I-C	14.61^a^±5,84	18.43^a. b^±7,17	23.51^a^±14,28	31.13^a. b. c^±15,59
I-1	14.52^a^±10,70	19.55^a. b^±15,61	20.81^a^±9,15	30.54^a. b^±16,94
I-2	13.69^a^±10,39	14.22^a^±8,73	17.81^a^±8,45	25.94^a^±10,24
G-C	17.01^a^±10,64	17.17^a. b^±7,19	23.69^a^±12,14	41.83^c^±19,39
G-1	12.75^a^±8.86	17.87^a. b^±9,06	24.44^a^±11,16	34.99^a. b. c^±8,76
G-2	11.96^a^±6,23	14.44^a^±8,82	22.44^a^±17,71	34.74^a. b. c^±15,32

DNA fragmentation index values immediately after thawing (sDFI 0) and after 4 (sDFI 4), 6 (sDFI 6) and 24 h (sDFI 24) of incubation. Treatments: *C*,standard control centrifugation, *1*, colloidal centrifugation Protocol 1, *2*, colloidal centrifugation Protocol 2, *B*, Botu-Crio®, *I*, InraFreeze®, *G*, Gent®. Letters in superscript represent significant differences between extenders (p<0.05) at each time point.

A comparison of the sDFI values at different incubation times between the nine treatments (Table 2) showed no significant difference between them at 0 and 6 h. However, at 4 h of incubation Protocol 2 was associated with a trend towards lower sDFI values (less DNA fragmentation); indeed significant differences (p<0.05) were detected between the I-2 treatment and the B-C treatment (sDFI 14.22 and 22.16 respectively), and between the G-2 treatment and the B-C treatment (sDFI 14.44 and 22.16 respectively).

At 24 h of incubation, the InraFreeze® extender tended to improve the sDFI results for all centrifugation protocols, with significant differences (p<0.05) seen between the I-2 and B-C treatments (sDFI 25.94 compared to 39.40), between the I-2 and G-C treatments (sDFI 25.94 compared to 41.83), and between the I-1 and G-C treatments (sDFI 30.55 compared to 41.83).

Irrespective of the centrifugation protocol followed, the sDFI values obtained at 6 h of incubation at 37°C were significantly (p<0.05) lower with InraFreeze® extender than with Botu-Crio® extender (Table 3). At 24 h, the sDFI values obtained with the InraFreeze® extender were lower than those obtained with either Botu-Crio® or Gent® extender.

**Table 3:** Average DNA fragmentation associated with the use of the different freezing extenders (means±SD)

**Extender**	**sDFI 0**	**sDFI 4**	**sDFI 6**	**sDFI 24**
Botu-Crio®	13,82^a^±8,57	18,78^a^±8,76	25,74^b^±11,10	36,08^b^±12,70
InraFreeze®	14,27^a^±9,07	17,40^a^±11,15	20,77^a^±12,74	29,18^a^±14,39
Gent®	13,91^a^±8,88	16,49^a^±8,36	23,52^a, b^±13,71	37,19^b^±15,20

DNA fragmentation index average values immediately after thawing (sDFI 0) and after 4 (sDFI 4), 6 (sDFI 6) and 24 h (sDFI 24) of incubation. Letters in superscript represent significant differences between extenders (p<0.05) at each time point.

Table 4 shows the sDFI values at different incubation times obtained with the different centrifugation protocols irrespective of the extender used. Both Protocol 1 and 2 returned significantly (p<0.05) lower sDFI values at 0 h than the SCC protocol. At 4 h, only Protocol 2 returned significantly lower sDFI values than the SCC protocol. No significant differences were found between any of the three centrifugation protocols at 6 or 24 h of incubation, although the values returned by Protocol 2 tended to be lower.

**Table 4:** Average DNA fragmentation values associated with the different centrifugation protocols (means±SD)

**Centrifugation Protocol**	**sDFI 0**	**sDFI 4**	**sDFI 6**	**sDFI 24**
Standard control centrifugation	21.50^b^±9,54	24.31^b^±8,97	24.74^a^±13,91	37.12^a^±16,56
Protocol 1	13.50^a^±8,85	17.83^a.b^±10,79	24.17^a^±11,41	33.90^a^±12,40
Protocol 2	12.50^a^±7,66	15.59^a^±8,35	21.19^a^±12,41	31.53^a^±13,86

DNA fragmentation index average values immediately after thawing (sDFI 0) and after 4 (sDFI 4), 6 (sDFI 6) and 24 h (sDFI 24) of incubation. Letters in superscript represent significant differences between extenders (p<0.05) at each time point.

## Discussion

The present sDFI results show variability between the donor stallions in terms of their post-thaw sperm DNA integrity. This is not surprising given the individual variability commonly seen in stallion sperm [2,3,32]. Horses have undergone selection primarily with pedigree in mind or for their ability to provide a particular type of service. In some cases, however, this has led to reduce fertility [9,15,20,23,33]. In the present work, the range of post-thaw sDFI values immediately after thawing (6.55-22.46%) recorded is similar to that reported by López-Fernández et al. [9] (4.2-26.3%).

Individual variability was observed in the DNA fragmentation dynamics recorded, as reported earlier by López-Fernández et al. [9]. In the present study, the sDFI values (i.e., DNA fragmentation levels) increased significantly with incubation time, as reported by other authors [6,9,34]. The largest increase occurred between 6 h and 24 h of incubation, not between 1 h and 6 h as described by López-Fernández et al. [9]. This may be significant since there is increasing evidence that the integrity of the sperm chromatin at the actual time of fertilization influences embryo survival [9]; poor sperm DNA integrity may account for some of the infertility commonly thought to lie with mares.

Hoogewijs et al. [28] obtained an average sDFI immediately after thawing of around 12% in colloidally-selected stallion sperm samples cryopreserved with Botu-Crio®, while Cortés-Gutiérrez et al. [35] reported values of 18-22% in non-colloidally-selected donkey sperm samples frozen with Gent®. The present results are consistent with the findings of these authors (13.24% for B-1, 11.86% for B-2 and 17.1% for G-C), although different DNA integrity evaluation techniques were used (SCSA, N-Comet Assay and the sperm chromatin dispersion test respectively).

No differences were seen in the sDFI between any of the nine treatments immediately after thawing. However, the use of InraFreeze® was associated with a trend towards the improvement of the sDFI values, especially at 24 h of incubation; indeed significant differences (p<0.05) were seen between I-2 and B-C, I-2 and G-C, and I-1 and G-C.

Irrespective of the centrifugation protocol followed, InraFreeze® significantly reduced the sDFI values at 6 h compared to Botu-Crio®, and at 24 h of incubation compared to both Botu-Crio® and Gent®. Some authors report that egg yolk-based extenders better protect chromatin structure than skimmed milk-based extenders, at least in bovine sperm [36]. Carvalho et al. [37], in contrast, report the extender employed to have no influence on DNA integrity. However, it should be noted that, in these two studies, the sDFI was analysed immediately after thawing; no post-thaw incubation study was undertaken - and DNA integrity would be most strongly affected during such an incubation period. The precise mechanism by which egg yolk aids in the protection of spermatozoa during the freeze-thaw process is unknown [38], but low density lipoproteins present in egg yolk plasma are widely presumed to be the cryoprotective agent [38,39]. Further, there is increasing evidence that cryoprotective antagonists may exist in other egg yolk fractions [38,39]. The above may explain the better sDFI values obtained with InraFreeze®, which contains only egg yolk plasma rather than whole egg yolk as in Botu-Crio® and Gent®. Replacing egg yolk with sterilized egg yolk plasma might remove a potential source of cryoprotective antagonists [39].

In the present work, single layer colloidal centrifugation prior to cryopreservation (Protocols 1 and 2) led to significantly lower (p<0.05) sDFI values than the SCC protocol immediately after thawing. These data are consistent with those obtained by other authors for human sperm [40,41], fresh equine semen [19-21,25], cooled equine semen [10] and cryopreserved equine semen subjected to colloidal centrifugation before freezing [28,29] or after thawing [17,29]. However, these authors performed no DNA fragmentation dynamics analysis.

The present results showed the I-2 and G-2 treatments to return significantly lower sDFI values than the B-C treatment at 4 h of incubation. At 24 h of incubation, Protocol 2 was associated with the best sDFI values for all three extenders. Crespo et al. [6] found no differences in sDFI values at time 0 of incubation between colloidal, standard or non- centrifuged equine fresh sperm samples, but as we found, after incubation at 37°C samples subjected to colloidal centrifugation exposed lower sDFI values than non-selected sperm both for fresh and cooled equine semen, what can be related to higher longevity in the selected sperm. Macías García et al. [42] have recently proved that colloidal centrifugation selects a spermatozoa subpopulation that clearly responds differently to osmotic shock, which could better withstand cooling procedures.

Sperm yield obtained from SCC was superior to what is usually described in the literature for simple centrifugation (400–600 g), where average losses are 20-25% [20,31] However, average sperm yields were similar to those obtained by Hoogewijs et al. [28] for colloidal centrifugation (57.79% vs. 50.9%) protocols. Hoogewijs et al. [28] reported colloidal centrifugation prior to cryopreservation to be associated with lower sperm yields than standard centrifugation. However, we agree with the theory of these authors that indicate that these lower sperm yields might be offset if colloidally-selected sperm better withstood cryopreservation, thus reducing the sperm dose required to guarantee conception.

In the present work most sperm DNA damage occurs during incubation, not in the first minutes after thawing, in concordance with other authors [6,9]. This fact remarks the importance of the use of the dynamic form of the SDF assay for evaluating ex vivo procedures [6]. sDFI values were significantly lower immediately after thawing and remained so in the colloidally-selected samples during the first four hours of incubation (24.31 for the SCC protocol vs. 15.59 for Protocol 2). Although there were no significant differences in sDFI values between the SCC protocol and Protocol 1, in the SCC protocol DNA damage occurred more quickly and was more intense. Crespo et al. [6], López-Fernández et al. [9] and Cortés-Gutiérrez et al. [11] indicate that slower DNA fragmentation dynamics may be associated with greater sperm viability in the female genital tract. The results suggest that colloidal centrifugation before cryopreservation would be beneficial in terms of spermatic survival after insemination with frozen-thawed sperm, although further studies are necessary to confirm a possible relation between our “*in vitro*” observations and “in vivo” fertility.

## Conclusion

Single layer colloidal centrifugation performed with extended or raw semen prior to cryopreservation reduces DNA fragmentation during the first four hours after thawing (while incubating at 37°C). Further studies are needed to determine the influence of freezing extenders on equine sperm DNA fragmentation dynamics.

## Abbreviations

ROS: Reactive Oxygen Species; SCC: C, Standard Control Centrifugation; 1: Colloidal centrifugation Protocol 1; 2: Colloidal centrifugation Protocol 2; sDFI: Sperm DNA Fragmentation Index; B: Botu-Crio®; I: Inrafreeze®; G: Gent®.

## Competing interests

The authors declare that they have no competing interests.

## Authors’ contributions

The study was conceived by CS conceived the study and together with FC and JG participated in its design and coordination. LG-C, AF and FC performed the semen collection, processing of samples for centrifugation and cryopreservation, and helped by MAR, JG and CS performed the sDFI dynamic assay. LG and CS did the statistical analysis and with FC interpreted the data. The manuscript was written by LG-C helped by CS. AF, MAR, FC and JG revised it critically. All authors have read and approved the final version of the manuscript.
